# Associations between Physical and Cognitive Doping – A Cross-Sectional Study in 2.997 Triathletes

**DOI:** 10.1371/journal.pone.0078702

**Published:** 2013-11-13

**Authors:** Pavel Dietz, Rolf Ulrich, Robert Dalaker, Heiko Striegel, Andreas G. Franke, Klaus Lieb, Perikles Simon

**Affiliations:** 1 Department of Sports Medicine, Rehabilitation and Disease Prevention, Johannes Gutenberg University, Mainz, Germany; 2 Department of Sports Medicine, Eberhard Karls University Tuebingen, Tuebingen, Germany; 3 Department of Psychiatry and Psychotherapy, University Medical Center, Mainz, Germany; 4 Department of Psychology, Eberhard Karls University Tuebingen, Tuebingen, Germany; University of Bath, United Kingdom

## Abstract

**Purpose:**

This study assessed, for the first time, prevalence estimates for physical and cognitive doping within a single collective of athletes using the randomized response technique (RRT). Furthermore, associations between the use of legal and freely available substances to improve physical and cognitive performance (enhancement) and illicit or banned substances to improve physical and cognitive performance (doping) were examined.

**Methods:**

An anonymous questionnaire using the unrelated question RRT was used to survey 2,997 recreational triathletes in three sports events (Frankfurt, Regensburg, and Wiesbaden) in Germany. Prior to the survey, statistical power analyses were performed to determine sample size. Logistic regression was used to predict physical and cognitive enhancement and the bootstrap method was used to evaluate differences between the estimated prevalences of physical and cognitive doping.

**Results:**

2,987 questionnaires were returned (99.7%). 12-month prevalences for physical and cognitive doping were 13.0% and 15.1%, respectively. The prevalence estimate for physical doping was significantly higher in athletes who also used physical enhancers, as well as in athletes who took part in the European Championship in Frankfurt compared to those who did not. The prevalence estimate for cognitive doping was significantly higher in athletes who also used physical and cognitive enhancers. Moreover, the use of physical and cognitive enhancers were significantly associated and also the use of physical and cognitive doping.

**Discussion:**

The use of substances to improve physical and cognitive performance was associated on both levels of legality (enhancement vs. doping) suggesting that athletes do not use substances for a specific goal but may have a general propensity to enhance. This finding is important for understanding why people use such substances. Consequently, more effective prevention programs against substance abuse and doping could be developed.

## Introduction

A growing concern in today’s society is the consumption of substances to increase physical or cognitive performance [1], [2], [3], [4]. For example, the use of drugs such as anabolic steroids in professional sports has long been a concern. In order to combat physical doping in professional sports, the World Anti-Doping Agency (WADA) annually lists banned substances [5]. However, physical doping is not only observed in elite athletes [4] but also in recreational athletes [6], [7], [8], [9]. Especially in fitness sports, high prevalences, between 5 - 20%, have been revealed for the use of physical performance-enhancing substances [3], [7], [8], [10], [11], [12]. Besides illicit or banned drugs, athletes also consume legal and freely available substances such as analgetics, caffeine, and other ergogenic aids (e.g. creatine, vitamins, minerals, carbohydrates, proteins), which may also improve physical performance [9], [13], [14], [15], [16]. It is of particular concern that these nutritional supplements have been shown to fail tests of safety, purity, and quality of ingredients and may contain prohibited substances [17], [18], [19]. In order to distinguish between these two levels of substance consumption, we use the term “physical doping” to refer to the former case, that is, the intake of illicit or banned substances to improve physical performance in sports, whereas the expression “physical enhancement” will be used to refer to the intake of legal or freely available substances for improving sports performance (Table 1).

**Table 1:** Examples of legal and illicit substances that produce effects on the body and cognitive functions.

Legality	Effect of Substance	
	*Physical*	*Cognitive*
*Enhancement* (legal or freely available substances)	e.g. creatine, vitamins, minerals,carbohydrates, proteins	e.g. caffeinated drinks, gingko biloba
*Doping* (illicit or banned substances/illicit substancesor pharmaceuticals)	e.g. anabolic androgenic steroids, humangrowth hormones, erythropoietin	e.g. modafinil, methylphenidate, antidepressants, beta-blockers

Besides the use of substances and methods to improve physical performance in sports, there is also a growing trend in our society to improve cognitive functions such as memory, attention, learning performance, or mood by the intake of substances [1], [20], [21]. Cognitive doping can include illicit substances (e.g. cocaine) and prescription drugs (pharmacological neuroenhancement) such as stimulants (e.g. methylphenidate and amphetamines), antidepressants, beta-blockers, or modafinil [21], [22], [23], which are primarily designed and used for the treatment of diseases. Prevalences for the use of such cognitive-enhancing substances range from 1.2% to 35% among German and American students [1], [20], [21], [24], [25], [26], and are estimated to be 20% among readers of the journal ‘Nature’ [2], 19.9% among surgeons [27], and 5% among office workers in Germany [28]. Besides illicit and prescription drugs, the use of legal and freely available substances such as ginkgo biloba or caffeinated drinks (e.g. coffee, energy drinks) are also a matter of debate although their ergogenic potential is still unknown [29], [30], [31], [32], [33]. As before, we distinguish between “cognitive doping” and “cognitive enhancement” depending on whether the substances for improving cognitive functions are illicit or only available in a pharmacy (Table 1).

Although the intake of substances to improve physical and cognitive functions appears to be widespread, we are not aware of a study that has examined whether the use of substances for each function is related. For example, several researchers have suggested that the use of nutritional supplements provides a gateway [34] to doping in sports [35], [36]. In other words, athletes who use physical enhancers may be especially prone to physical doping. Likewise, it seems reasonable to assume that a similar gateway exists from cognitive enhancement to cognitive doping. Moreover, some people may have a propensity to consume substances to improve not only their cognitive performance but also their physical performance. Consequently, there should not only be an association between doping and enhancement but also between cognitive enhancement and physical enhancement, and between cognitive doping and physical doping. It is the major aim of this survey to evaluate these hypothesized associations in a collective of about 3,000 full-distance and half-distance triathletes. The decision to perform the survey in a collective of triathletes is based on the fact that on the one hand, triathlon, and especially full- and half-distance triathlon, is an ultra-endurance sport and on the other hand, it is a rather expensive sport (e.g. swim suits, shoes, bikes, nutrition, travel expenses to competitions, and starter fees must all be financed by the athletes). Therefore, the use of substances to enhance physical performance in training and competition as well as the use of cognitive-enhancing substances to enhance workplace performance seem to be plausible.

The prevalence for the use of legal and freely available substances for physical and cognitive enhancement was assessed by an anonymous questionnaire. This questionnaire also adopted the randomized response technique (RRT) [37] to obtain more valid prevalence estimates of the use of illicit and banned substances to improve physical performance (i.e. physical doping) and the use of illicit substances and pharmaceuticals to improve cognitive performance (i.e. cognitive doping) within the same collective [38].

## Materials and Methods

### Sample, Survey Procedure, and Ethics

Ethical approval to conduct this study was obtained by the Eberhard Karls University of Tuebingen Ethics Committee. Written consent to participate in the survey was given by the participants within the questionnaire.

The study surveyed participants of the long-distance triathlons (3.8 km–180.2 km–42.2 km) in Frankfurt (European Championship) and Regensburg and also the half-distance triathlon in Wiesbaden (European Championship 70.3). A short self-report paper-and-pencil questionnaire was distributed to the participants during the registration procedure in the race-office on Thursday, Friday, and Saturday prior to the competition which took place on Sunday. Professional athletes (*N* = 134; 2.1%) did not pass through the race-office and thus were not included in this survey. Therefore, the present results are representative of recreational triathletes. All registered athletes were informed in advance about the survey by mail. After athletes had completed the questionnaire, they were asked to drop the filled-in questionnaire into a black box and return the clipboard and the pen to the researchers. By using this box, we wanted to emphasize the anonymity of this survey. To further increase the level of anonymity of this survey, any personal information within the questionnaire (e.g. name, date of birth, best result in competition) was avoided. In addition, the term “doping” was circumvented in the questionnaire in order to reduce any reluctance to complete the survey. Instead “doping”, we used the terms “physical enhancement” and “cognitive enhancement”. Both German and English versions of the questionnaire were available.

### Questionnaire

At the beginning of the questionnaire, athletes were informed about the aim of the survey and that participation was anonymous and voluntary. Within the questionnaire, the RRT was used to assess the 12-month prevalence of illicit or banned substance use with the exclusive purpose of enhancing physical performance (i.e. physical doping) as well as the use of illicit drugs or pharmaceuticals with the exclusive purpose of enhancing cognitive performance (i.e. cognitive doping). The 12-month prevalence rather than the lifetime prevalence was examined so that the results can be compared with follow-up surveys. Caffeine tablets but not caffeine per se (e.g. a large cup of coffee) were included in the definition of cognitive doping because in Germany (in contrast to the U.S.) these tablets can only be bought in pharmacies and not in supermarkets or drug-stores. To our mind, the consumption of caffeine tablets differs markedly from the consumption of a cup of coffee; the latter merely increases the level of alertness. Some information was assessed with two closed-ended questions: gender (male/female), A-level – final qualification for university entrance (yes/no), do you train with a structured training plan (yes/no), behavior in case of pain during training (continue/take a pause), 12-month prevalence for the use of legal and freely available substances for physical (yes/no), and cognitive enhancement (yes/no). Further questions required information about age, height, weight, years of training, hours of training per week, kilometers per week cycling/running/swimming.

The complete question for assessing the use of legal substances was: “Have you used legal or freely available substances with the purpose of enhancing your cognitive performance (e.g. ginkgo biloba)/to enhance your physical performance (e.g. creatine, colostrum) during the last 12 months?” The four response categories to this question were: (a) Yes, to improve cognitive performance, (b) Yes, to improve physical performance, (c) Yes, to improve both cognitive and physical performance, and (d) No consumption of such substances.

### Randomized Response Technique (RRT)

Prevalence for physical doping and cognitive doping were assessed by the unrelated question technique [39]. When administering this RRT in our previous studies, participants were asked to draw a card of their choice from a deck of 20 cards. Seventy-five per cent of the cards contained the sensitive question and 25% contained a non-sensitive question [3], [4]. Since the interviewers cannot know which card the participant has drawn to answer, the participants can reply honestly without compromising themselves. This procedure was emulated by using a paper-and-pencil version of the randomization process which allowed the RRT procedure to be included in the present questionnaire [1], [27]. The emulated RRT procedure to assess the prevalence of physical and cognitive doping is presented in Table 2 and Table 3.

**Table 2:** RRT procedure to assess physical doping.

	Please consider a certain birthday (yours, your mother’s, etc.). Is this birthday in the first third of a month (1^st^ to 10^th^ day)? If yes, please proceed to Question A; if no, please proceed to Question B.	
Question A	Is this birthday in the first half of the year (prior to the first of July)?	
Question B	Have you used substances which can only be prescribed by a doctor, are available in a pharmacy, or can be bought on the black market (e.g. anabolic steroids, erythropoietin, stimulants, growth hormones) to enhance your physical performance during the last 12 months?	
	Note that only you know which of the two questions you will answer	
	Yes	No

**Table 3:** RRT procedure to assess cognitive doping.

	Please consider another birthday (your friends, your father’s, etc.). Is this birthday in the second third of a month (11^st^ to 20^th^ day)? If yes, please proceed to Question A; if no, please proceed to Question B.	
Question A	Is this birthday in the first half of the year (prior to the first of July)?	
Question B	Have you used substances which can only be prescribed by a doctor, are available in a pharmacy, or can be bought on the black market (e.g. caffeine tablets, stimulants, cocaine, methylphenidate, modafinil, beta-blockers) to enhance your cognitive performance during the last 12 months?	
	Note that only you know which of the two questions you will answer	
	Yes	No

Accordingly, the probability of receiving the neutral question A is 32.9% (120/365.25), whereas the probability of receiving the sensitive question B is 67.1% (245.25/365.25). The proportion of ‘yes’ responses with respect to the sensitive questions (i.e., the prevalence estimate 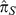) is computed from proportion 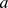 of total ‘yes’ responses in the sample by using the following formula 
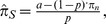
 where 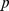 denotes the probability of receiving the sensitive question (in our case 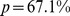). The probability of answering the neutral question with ‘yes’ is 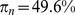 (181.25/365.25). A 95% confidence interval (CI) for the unknown prevalence is obtained from the sampling variance where 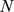 denotes the sample size: 
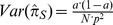
.

### Statistics

Prior to this survey, a statistical power analysis [40] was conducted to determine the necessary sample size for obtaining reliable RRT estimates. The null hypothesis of this power analysis assumes that the true prevalence is equal to zero, that is, 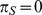. To detect an overall prevalence of at least 6% with a statistical power of 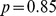, the sample size n should be at least equal to 650. According to previous competitions, only one quarter of the athletes was female. Consequently, a minimum of 2,600 questionnaires were required to ensure that 650 female athletes would complete the questionnaire so that the RRT would yield meaningful results even for this subsample.

Regarding the analysis of physical and cognitive enhancement, percentages and relative percentages were computed. Continuous variables were analyzed using the non-parametric Mann-Whitney-U-Test and categorical variables using Pearsońs Chi-Square-Test. P-values were corrected by the method of Bonferroni by multiplying each p-value with the factor 16 because 16 tests were performed for each of the two dependent variables ‘physical enhancement’ and ‘cognitive enhancement’ (one for each predictor variable). Binary logistic regressions using backward stepwise elimination were employed to predict legal substance use for physical and cognitive enhancement. Continuous variables were dichotomized. Odds ratios (OR) and 95% confidence intervals (CI) were adjusted for age, gender, and Body-Mass-Index (BMI). Finally the bootstrap method was used to compute 95% confidence intervals for differences on the prevalence estimates [41]. Each sampling distribution of the simulated differences was based on 50,000 bootstrap samples. Statistical analyses were carried out using SPSS for Windows 19.0 and MATLAB 2010.

## Results

### Response Rate, Biographical Data, and Training Characteristics of the Participants

2,997 questionnaires were distributed and 2,987 (99.7%) were returned. From these returned questionnaires, 31.1% (*N* = 934) were collected at the European Championship in Frankfurt, 32.6% (*N* = 974) in Regensburg, and 36.1% (*N* = 1,079) at the European Championship 70.3 in Wiesbaden. The percentage of responses to the two RRT questions were 90.5% (*N* = 2,702) for physical doping and 91.6% (*N*2,737) for cognitive doping. 5.4% of the participants (*N* = 162) completed the first part of the questionnaire but not the two sensitive RRT questions. Most athletes were male (*N* = 2,576, 87.3%) and with mean age of 39.5 years (*SD*: ±9.2; range 18–79). Table 4 contains the biographical data of all athletes and Table 5 information about their training schedule.

**Table 4:** Biographical data of the participants.

**Questionnaires distributed**	N = 2997
**Participants (total)**	N = 2987
**Response rate**	99.7%
**Location**	
Frankfurt (European Championship)	31.3% (N = 934)
Regensburg	32.6% (N = 974)
Wiesbaden (European Championship 70.3)	36.1% (N = 1079)
**Gender**	87.3% male (N = 2576)
	12.7% female (N = 376)
**Age**, years (mean; SD)	18–79 (39.5 years ±9.2)
**Height**, cm (mean; SD)	146–207 (179.1 cm ±8.2)
**Weight**, kg (mean; SD)	46–126 (74.4 kg ±9.6)
**BMI**, kg/m^2^ (mean; SD)	16.9–42.5 (23.1 kg/m^2^±2.2)
**A-level**	65.3% yes (N = 1742)
	34.7% no (N = 925)
**Questionnaire version**	65.5% German (N = 1956)
	34.5% English (N = 1031)

(cm = centimeters; kg = kilograms; m = meters; SD = standard deviation).

**Table 5:** Training characteristics.

**Years of training**, years (mean; SD)	0–52 (9.7 years ±8.2)
**Hours of training/week**, hours (mean; SD)	1–65 (13.2 hours ±5.1)
**Kilometers bike/week**, km (mean; SD)	7–800 (190.0 km ±94.6)
**Kilometers running/week**, km (mean; SD)	0–150 (41.8 km ±18.6)
**Kilometers swimming/week**, km (mean; SD)	0–80 (6.4 km ±4.5)
**Structured training plan**	65.6% yes (N = 1953)
	34.4% no (N = 1023)
**Behavior in case of pain during training**	61.0% take a pause(N = 1795)
	39.0% continue (N = 1031)

(kg = kilograms; km = kilometers; SD = standard deviation).

### Physical and Cognitive Enhancement

Table 6 summarizes the response frequencies concerning the use of enhancers. These results show that 14.1% and 5.8% of all athletes reported the use of such substances to enhance physical and cognitive performance, respectively, during the last 12 months. From the total number of all athletes, 3.9% admitted using both physical and cognitive enhancers during the last 12 months.

**Table 6:** 12-month prevalences for the use of legal and freely available substances.

Physical enhancement only	10.2% (N = 290)
Cognitive enhancement only	1.9% (N = 55)
Both	3.9% (N = 110)
None	84.0% (N = 2396)

Table 7 contains 2 by 2 contingency tables to assess potential associations between physical or cognitive enhancement with other category variables of the questionnaire such as gender, training plan, and so on. The left column of Table 7 shows how physical enhancement is related to these variables. First, cross-tabulation of physical and cognitive enhancement revealed a significant association (*χ^2^* = 402.3; *df = *1; *p*<.001; see Table 7, left column). Specifically, athletes who reported the use of physical enhancers also tended to report the use of cognitive enhancers (φ = 0.38). Second, physical enhancement was slightly yet significantly associated with training plan (*χ^2^* = 12.4; *df = *1; *p*<.001); more athletes that had a structured training plan reported using physical enhancers than athletes without such a plan (φ = 0.07). Finally, the use of physical enhancers differed between athletes who paused after the occurrence of pain during training and those who did not (*χ^2^* = 24.5; *df = *1; *p*<.001); athletes who paused were less likely to use physical enhancers than those who did not pause (φ = −0.09). Besides these cross-tabulation results, further analyses showed that the use of physical enhancers was related to a higher level of sports-specific training (lower left part of Table 7), that is, athletes who use physical enhancers also tend to train more in contrast to those athletes who do not use physical enhancers. The percentage of physical enhancement was not significantly modulated by location of sports event, gender, A-level, age, height, BMI, or years of training.

**Table 7:** Associations between the two dependent variables ‘physical enhancement’ and ‘cognitive enhancement’ and each predictor variable.

	Physical enhancement			Cognitive enhancement			
	Yes	No	*p-value*	Yes	No	*p-value*	
**Cognitive enhancement**							
Yes	N = 110 (3.9%)	N = 55 (1.9%)	a.000***				
	**rel. 66.7%**	**rel. 33.3%**					
No	N = 290 (10.2%)	N = 2396 (84.0%)					
	**rel. 10.8%**	**rel. 89.2%**					
**Location**							
Frankfurt	N = 128 (4.5%)	N = 749 (26.3%)	a *n.s.*	N = 56 (2.0%)	N = 821 (28.8%)	a *n.s*	
	**rel. 14.6%**	**rel. 85.4%**		**rel. 6.4%**	**rel. 93.6%**		
Regensburg	N = 132 (4.6%)	N = 802 (28.1%)		N = 58 (2.0%)	N = 876 (30.7%)		
	**rel. 14.1%**	**rel. 85.9%**		**rel. 6.2%**	**rel. 93.8%**		
Wiesbaden	N = 140 (4.9%)	N = 900 (31.3%)		N = 51 (1.8%)	N = 989 (34.7%)		
	**rel. 13.5%**	**rel. 86.5%**		**rel. 4.9%**	**rel. 95.1%**		
**Questionnaire**							
English	N = 141 (4.9%)	N = 803 (28.2%)	a *n.s.*	N = 78 (2.7%)	N = 866 (30.4%)	a.001**	
	**rel. 14.9%**	**rel. 85.1%**		**rel. 8.3%**	**rel. 91.7%**		
German	N = 259 (9.1%)	N = 1648 (57.8%)		N = 87 (3.1%)	N = 1820 (63.8%)		
	**rel. 13.6%**	**rel. 86.4%**		**rel. 4.6%**	**rel. 95.4%**		
**Gender**							
Female	N = 47 (1.7%)	N = 319 (11.3%)	a *n.s.*	N = 19 (0.7%)	N = 347 (12.3%)	a *n.s.*	
	**rel. 12.8%**	**rel. 87.2%**		**rel. 5.2%**	**rel. 94.8%**		
Male	N = 349 (12.4%)	N = 2103 (74.6%)		N = 144 (5.1%)	N = 2308 (81.9%)		
	**rel. 14.2%**	**rel. 85.8%**		**rel. 5.9%**	**rel. 94.1%**		
**A-level**							
Yes	N = 238 (9.3%)	N = 1435 (56.0%)	a *n.s.*	N = 96 (3.7%)	N = 1577 (61.6%)	a *n.s.*	
	**rel. 14.2%**	**rel. 85.8%**		**rel. 5.7%**	**rel. 94.3%**		
No	N = 117 (4.6%)	N = 771 (30.1%)		N = 45 (1.8%)	N = 843 (32.9%)		
	**rel. 13.2%**	**rel. 86.8%**		**rel. 5.1%**	**rel. 94.9%**		
**Training plan**							
Yes	N = 293 (10.3%)	N = 1568 (55.2%)	a.007**	N = 128 (4.5%)	N = 1733 (61.0%)	a.012*	
	**rel. 15.7%**	**rel. 84.3%**		**rel. 6.9%**	**rel. 93.1%**		
No	N = 107 (3.8%)	N = 873 (30.7%)		N = 37 (1.3%)	N = 943 (33.2%)		
	**rel. 10.9%**	**rel. 89.1%**		**rel. 3.8%**	**rel. 96.2%**		
**Behavior in case of pain**							
Pause	N = 195 (6.9%)	N = 1527 (54.4%)	a.000***	N = 73 (2.6%)	N = 1649 (58.7%)	a.000***	
	**rel. 11.3%**	**rel. 88.7%**		**rel. 4.2%**	**rel. 95.8%**		
Continue	N = 195 (6.9%)	N = 891 (31.7%)		N = 88 (3.1%)	N = 998 (35.5%)		
	**rel. 18.0%**	**rel. 82.0%**		**rel. 8.1%**	**rel. 91.9%**		
**Age**, years; median (quartil 25; 75)	38 (32, 45)	40 (33, 46)	b *n.s.*	39 (31, 45)	40 (33, 46)	b *n.s.*	
**Height**, cm; median (quartil 25; 75)	180 (174, 185)	180 (174, 184)	b *n.s.*	179 (173, 184)	180 (175, 184)	b *n.s.*	
**Mass**, kg; median (quartil 25; 75)	74 (69, 80)	74 (69, 80)	b *n.s.*	74 (68, 80)	74 (69, 80)	b *n.s.*	
**BMI**, kg*m^−2^; median (quartil 25; 75)	23 (22, 24)	23 (22, 24)	b *n.s.*	23 (21, 24)	23 (22, 24)	b *n.s.*	
**Years of training**, years; median (quartile 25; 75)	7 (4, 14)	7 (4, 14)	b *n.s.*	7 (3, 14)	7 (4, 13)	b *n.s.*	
**Hours/week**, hours; median (quartil 25; 75)	14 (10, 17)	12 (10, 15)	b.000***	15 (11, 18)	12 (10, 15)	b.005**	
**Km/week bike**, km; median (quartil 25; 75)	200 (150, 250)	180 (120, 250)	b.000***	200 (150, 250)	200 (120, 250)	b *n.s.*	
**Km/week running**, km; median (quartil 25; 75)	42 (30, 55)	40 (30, 50)	b.001**	40 (30, 50)	40 (30, 50)	b *n.s.*	
**Km/week swimming**, km; median (quartil 25; 75)	6 (4, 9)	5 (4, 8)	b.000***	6 (5, 10)	5 (4, 8)	b.000***	

Levels of significance: *p*<.05*; *p*<.01**; *p*<.001***; p-values corrected by the method of Bonferroni.

a Pearsońs Chi-Square-Test.

b Mann-Whitney-U-Test (Gaussian distribution not fulfilled).

(cm = centimeter; kg = kilogram; m = meter; n.s. = not significant; SD = standard deviation).

The right column of Table 7 contains the results of cross-tabulation with regard to cognitive enhancement. First, athletes who filled in the English questionnaire reported the use of cognitive enhancers more often than those who filled in the German questionnaire (*χ^2^* = 15.9; *df = *1; *p*<.001; φ = 0.07). Second, athletes with a training plan more often reported the use of cognitive enhancers than those without such a plan (*χ^2^* = 11.3; *df = *1; *p*<.001, φ = 0.06). Finally, athletes who paused after the occurrence of pain during training reported using physical enhancers less often than those who did not pause (*χ^2^* = 18.4; *df = *1; *p<*.001, φ = −0.08). In addition to these cross-tabulation results, other results show that athletes using cognitive enhancers also tend to train harder than those who do not use such enhancers, although this effect was only statistically reliable for the variables ‘training of hours per week’ and ‘kilometers of swimming per week’. The use of cognitive enhancers was not modulated by location of sport event, gender, A-level, age, height, BMI, or years of training.

In order to gain further information, separate logistic regression analyses were performed for the two dependent variables ‘physical enhancement’ and ‘cognitive enhancement’ (Table 8). All the afore mentioned significant variables were used as explanatory variables in two binary regression analyses using backward elimination. The best set of explanatory variables for predicting ‘physical enhancement’ were ‘cognitive enhancement’, ‘behavior in the case of pain’, and ‘kilometers of swimming per week’. By contrast, the best set of explanatory variables for predicting ‘cognitive enhancement’ were ‘physical enhancement’ and ‘language of the questionnaire’. The area under the receiver operating curve was.83 for the cognitive model and.70 for the physical model.

**Table 8:** Odds ratios for the dependent variables ‘physical enhancement’ and ‘cognitive enhancement’ and each predictor variable (stepwise, feed backward elimination).

Physical enhancement		Cognitive enhancement		
Predictor	Adjusted OR (95% CI)	Predictor	Adjusted OR (95% CI)	
Cognitive enhancement (no)	0.07*** (0.048–0.103)	Physical enhancement (no)	0.071*** (0.049–0.103)	
Behavior in case of pain (pause)	0.675** (0.528–0.862)	Questionnaire (German)	0.562** (0.385–0.819)	
Km per week bike (fewer)	0.75 (0.559–1.007)	Behavior in case of pain (pause)	0.724 (0.449–1.049)	
Km per week swimming (fewer)	0.742* (0.561–0.982)	Hours of training per week (fewer)	0.709 (0.472–1.065)	

Levels of significance: *p*<.05*; *p*<.01**; *p*<.001***.

OR adjusted for age, gender, and Body-Mass-Index.

(CI = confidence interval; OR = odds ratio).

### RRT Results for Physical and Cognitive Doping

In the total sample, the 12-month prevalence estimate for physical doping was 13.0% (CI: 10.5–15.4%). Table 9 shows how this estimate depends on other variables such as physical and cognitive enhancers. First, the estimated prevalence of physical doping was significantly higher when athletes reported the use of physical enhancers (20.6%) than when they reported not using such enhancers (11.4%). Bootstrapping revealed that the difference between these two estimates was significant (mean difference = 9.2%, SE = 3.8%, CI: 1.9–16.6%). Second, likewise, the estimated prevalence for physical doping was higher when athletes also used cognitive enhancers (23.1%) than when they did not use cognitive enhancers (12.0%), although this difference just failed statistical significance (mean difference = 11.1%, SE = 5.7%, CI: 0.0–22.3%). Third, the estimate was higher for male (13.7%) than for female athletes, although this difference was only marginally significant (mean difference = 5.6%, SE = 3.6%, CI: −1.5–12.6%). Fourth, although the prevalence rate was higher for athletes with a structured training plan than for those without such a plan, the difference was again only marginally significant (mean difference = 5.4%, SE = 3.5%, CI: −1.7–12.2%). Finally, although the estimated prevalence was lower for those athletes who paused in training after they experienced pain than those who did not, the observed difference was not significant (mean difference = −3.4%, SE = 2.6%, CI: −8.7–1.5%). Besides these results, the estimated 12-month prevalence of physical doping was clearly higher in athletes who competed in the European Championship in Frankfurt (19.8%; CI: 15.1–24.4) than in athletes who competed in Regensburg (10.3%; CI: 6.2–14.5) or Wiesbaden (9.7%; CI: 5.8–13.5).

**Table 9:** Estimated 12-month prevalence for physical doping by using RRT.

Population	‘Yes’	‘No’	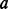	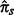	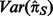	95% CI
**All athletes** (response rate = 90.5%)	676	2026	0.334	13.0%	0.00015	10.5–15.4
**Physical enhancement**						
‘Yes’	115	267	0.301	20.6%	0.00082	13.7–27.4
‘No’	542	1723	0.239	11.4%	0.00012	8.7–14.0
**Cognitive enhancement**						
‘Yes’	50	107	0.318	23.1%	0.00206	12.3–34.0
‘No’	607	1883	0.244	12.0%	0.00011	9.5–14.5
**Location**						
Frankfurt	245	583	0.296	19.8%	0.00056	15.1–24.4
Regensburg	202	667	0.232	10.3%	0.00046	6.2–14.5
Wiesbaden 70.3	229	776	0.228	9.7%	0.00039	5.8–13.5
**Gender**						
Male athletes	593	1733	0.255	13.7%	0.00018	11.1–16.3
Female athletes	75	271	0.217	8.0%	0.00109	1.5–14.5
**A-level**						
‘Yes’	409	1191	0.256	13.8%	0.00026	10.6–17.0
‘No’	205	630	0.246	12.3%	0.00049	7.9–16.6
**Structured training plan**						
‘Yes’	465	1306	0.263	14.8%	0.00024	11.8–17.9
‘No'	209	712	0.227	9.5%	0.00042	5.5–13.5
**Behavior in case of pain**						
Take a pause	395	1238	0.242	11.7%	0.00025	8.7–14.8
Continue	274	756	0.266	15.3%	0.00042	11.3–19.4

(CI = confidence interval).

The estimated 12-month prevalence for cognitive doping (i.e. use of illicit drugs or pharmaceuticals for cognitive enhancement) was 15.1% (CI: 12.7–17.6%) for all athletes. Table 10 reveals how this estimate is modulated by the variables mentioned before. First, athletes who used physical enhancers were more likely to also use cognitive doping substances than those who did not use physical enhancers (mean difference = 21.3%, SE = 4.0%, CI: 13.5–29.1%). Second, there was a strong positive relationship between the use of cognitive enhancers and cognitive doping substances (mean difference = 36.7%, SE = 6.1%, CI: 24.7–48.6%). Third, the estimate of cognitive doping was again numerically higher for males than for females, although this difference was again non-significant (mean difference = 4.9%, SE = 3.6%, CI: −2.4–11.9%). Fourth, and as before, the estimated doping prevalence did not significantly differ between athletes with a structured training plan and those without such a plan (mean difference = 0.8%, SE = 2.6%, CI: −4.4–5.9%). Finally, and in contrast to the previous analysis on physical doping, athletes who discontinued training after experiencing pain admitted significantly less often to cognitive doing compared with those who continued training (mean difference = −6.0%, SE = 2.6%, CI: −11.1– −0.85%). Moreover, the RRT estimates on cognitive doping did not significantly differ between the various locations of the sports events.

**Table 10:** Estimated 12-month prevalence for cognitive doping by using RRT.

Population	‘Yes’	‘No’	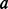	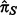	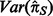	95% CI
**All athletes** (response rate = 91.6%)	724	2013	0.265	15.1%	0.00016	12.7–17.6
**Physical enhancement**						
‘Yes’	147	235	0.385	33.0%	0.00092	25.8–40.3
‘No’	555	1741	0.242	11.7%	0.00012	9.1–14.3
**Cognitive enhancement**						
‘Yes’	77	79	0.494	49.2%	0.00239	37.5–60.9
‘No’	625	1897	0.248	12.6%	0.00011	10.1–15.1
**Location**						
Frankfurt	226	609	0.271	16.0%	0.00052	11.5–20.5
Regensburg	242	643	0.274	16.4%	0.00050	12.1–20.8
Wiesbaden 70.3	256	761	0.252	13.2%	0.00041	9.2–17.1
**Gender**						
Male athletes	632	1726	0.268	15.6%	0.00018	13.0–18.3
Female athletes	82	267	0.235	10.7%	0.00114	4.1–17.3
**A-level**						
‘Yes’	430	1189	0.266	15.3%	0.00027	12.1–18.5
‘No’	207	638	0.245	12.2%	0.00049	7.9–16.5
**Structured training plan**						
‘Yes’	475	1310	0.266	15.4%	0.00024	12.3–18.4
‘No’	246	696	0.261	14.6%	0.00045	10.4–18.8
**Behavior in case of pain**						
Take a pause	413	1243	0.249	12.9%	0.00025	9.8–16.0
Continue	301	739	0.289	18.8%	0.00044	14.7–22.9

(CI = confidence interval).

In a final analysis, the responses for cognitive doping were cross-tabulated with those for physical doping (Table 11). We conjectured that these two response categories should be positively associated if athletes who use physical doping also tend to use cognitive doping and vice versa. It should be kept in mind, however, that much of the data that enter this analysis were in response to the non-sensitive questions in the RRT task. These responses can be considered random noise, which would simply lower the true association between responses to the sensitive doping questions. Nevertheless, if there is a latent association between cognitive and physical doping, one should expect a positive association despite the presence of this random noise. In fact, a Pearsońs chi-square-test revealed a clear positive association between cognitive and physical doping (*χ^2^* = 70.5; *df = *1; *p<*.001, φ = 0.16), supporting the notion that cognitive and physical doping are also strongly associated.

**Table 11:** Cross tabulation for the RRT answers concerning physical and cognitive doping.

Cognitive doping	Physical doping	
	Yes	No
Yes	N = 274 (10.2%)	N = 436 (16.3%)
No	N = 397 (14.8%)	N = 1571 (58.7%)

## Discussion

The present survey examined the use of substances to improve physical and cognitive performance in a large collective of recreational triathletes. Specifically, this study assessed the prevalence of using physical and cognitive enhancers (i.e. legal and freely available substances) as well as the prevalence of physical and cognitive doping (i.e. illicit or banned substances/pharmaceuticals) within the same sample. This design allowed us to examine whether the athletes who use substances to improve physical performance would also use substances to enhance cognitive performance and vice versa. To our knowledge this has not been addressed before.

The high response rate of 99.7% was achieved for several reasons. First, the study was performed during the registration procedure in the race-office, through which all recreational athletes had to pass and where they had enough time to complete the survey. Furthermore, the athletes were informed in advance by mail about the survey. Second, a black box was placed in the race-office where the athletes had to return the filled-in questionnaires. The use of this box emphasized anonymity to the participants, as there was a visual separation between the completed questionnaires and the researchers. The response rates for the RRT questions concerning physical and cognitive doping were 90.5% and 91.6%, respectively. 5.4% of the athletes stopped completing the questionnaire directly before the sensitive part and this may have somewhat biased the present estimates [42], [43]. Therefore, it is possible that the prevalences for physical and cognitive doping of 13.0% and 15.1% underestimated the true prevalences.

Logistic regression adjusted for age, gender, and BMI identified three variables that predicted the use of physical enhancers. Athletes who did not use cognitive enhancers, took a pause in response to pain during training, and completed fewer kilometers of swimming per week were less likely to use physical enhancers. The use of cognitive enhancers turned out to be the strongest predictor of physical enhancer use. This finding corroborates the results of a pilot study conducted in recreational marathon runners, which found that the use of prescription drugs for physical enhancement was significantly associated with the use of prescription drugs for cognitive enhancement and with continuing training in the case of pain [unpublished data, Dietz et al, under review]. Concerning training characteristics, in this pilot study only the number of kilometers for swimming, but not for running and cycling, predicted the use of legal physical enhancers. These pilot data also indicated a strong association between physical and cognitive enhancers, as well as between physical and cognitive doping, consistent with the present study. Some athletes may misuse these substances due to a general desire to improve one’s performance. In this context, we defined this type of improvement as a “general propensity to enhance”. Since the present study is the first one to assess substance use to enhance both physical and cognitive performance, future studies are required to examine this phenomenon in more detail.

Regarding the regression model for the use of cognitive enhancers adjusted for age, gender, and BMI, only the use of physical enhancers and the German version of the questionnaire were identified as predictor variables, indicating that athletes who completed the German version of the questionnaire had a lower relative risk of using legal cognitive enhancers. This result can be interpreted as evidence that foreign athletes, who completed the English version, are more likely to use legal and freely available substances than Germans athletes. On the other hand, it is possible that athletes who travel to other countries to compete in a triathlon need to earn enough money to finance these trips, and therefore have more disposable income to spend on substances.

The estimated 12-month prevalence for physical doping of 13.0% in this collective of recreational athletes is close to the estimated lifetime prevalence for physical doping in recreational fitness center users which was 12.5% [3]. Regarding the use of substances with the purpose of cognitive enhancement in athletes, to the best of our knowledge this is the first investigation assessing this item in athletes.

The prevalence of cognitive doping was higher than the prevalence of physical doping. At first sight, this seems to be paradoxical. The use of cognitive doping in athletes, however, is not forbidden and not associated with sanctions. In contrast to this, the use of physical doping is forbidden and the athletes are aware that doping testing is common practice in triathlon competitions, since the International Triathlon Union (ITU) [44] as well as the World Triathlon Cooperation (WTC) [45] insist on a strict anti-doping policy. In Frankfurt, Regensburg, and Wiesbaden doping tests were performed and financed by the organizer of the competitions. Consequently, athletes may either perceive the question regarding physical doping as especially sensitive, which may result in an underestimation of the true prevalence, or they may indeed not engage in physical doping as frequently as in cognitive doping. As in a previous article of our group [1], we included caffeine tablets in the definition of cognitive doping. This is because caffeine is not only a harmless flavouring agent but it is considered to be an effective and legal CE substance with pharmacological effects and side-effects. Therefore, caffeine is part of the 10^th^ International Classification of Diseases (ICD-10) and the 4^th^ Diagnostic and Statistical Manual of Mental Disorders (DSM-IV) as a psychoactive substance, among others (e.g. cocaine, alcohol, etc.) [30], [46]. Additionally, several clinical trials have demonstrated that caffeine is equally as effective as amphetamines and modafinil in some cognitive tests in healthy subjects [47], [48]. The difference between the use of caffeine tablets and other caffeinated products such as coffee is that the only motivation for taking caffeine tablets is to enhance alertness. This is in contrast to the various intentions that may underlie the intake of other caffeinated products Moreover, caffeine tablets can only be purchased in a pharmacy, in Germany.

The prevalence estimates of physical doping varied significantly across locations. The prevalence estimate of the European Championship in Frankfurt was significantly higher than the estimates of Regensburg and Wiesbaden. This result may be due to the fact that the European Championship in Frankfurt was of higher prestige than the competition in Regensburg, which was more regional. Additionally, in Frankfurt many more slots and qualification-points for the World Championship in Hawaii could be gained than in Regensburg. In Wiesbaden, in contrast to Frankfurt, only the half-distance triathlon was performed and therefore lower rates for physical doping seem plausible.

Concerning a potential gateway [34], [49] for physical doping, the prevalence estimate of physical doping was significant higher in athletes who also used physical enhancers. This association prompts the conclusion that the use of physical enhancers is a potential gateway to physical doping [35], [36]. The present data can only show an association between physical doping and physical enhancers. Hence, we do not know which type of performance enhancement was practiced first, the illicit or the legal one. To address this issue, Lentillon-Kaestner and colleagues (2010) [50] performed qualitative semi-structured interviews in a sample of young elite cyclists and highlighted that the use of nutritional supplements was the first step to physical doping. In non-elite sports, the same process seems possible but further studies are lacking. One meaningful difference between our survey and the surveys performed by the other groups supporting the gateway theory is the age of the participants. The interviews by Lentillon-Kaestner and colleagues (2010) [50] were performed in young (mean age 22.8 years) elite cyclists. The mean age of the collectives by Backhouse and colleagues (2011) [35] and Papadopoulos and colleagues (2006) [36] was 21.4 and 24.0 years, respectively. In contrast, the athletes who completed our survey were much older (mean age 39.7 years). In a previous survey by our group we found no correlation between physical doping and nutritional supplement use among professional master athletes with a mean age of 52.8 years [9]. Since in the present survey we assessed the 12-month prevalence rather than the lifetime prevalence, it seems plausible that a potential gateway may also apply to older athletes. Since doping testing is common practice in triathlon competitions, it may also be possible that physical doping is only practiced during training, while during competitions, athletes mostly use physical enhancers.

Concerning a potential gateway for cognitive doping, the prevalence estimate for cognitive doping was significantly higher in athletes who used cognitive enhancers than those who did not. Since we do not know which type of substance was used first by the athletes – legal and freely available or illicit substances – these data do not strongly support the gateway theory [34], [49] that the use of cognitive enhancers is the first step for cognitive doping. Nonetheless, a potential gateway theory for cognitive enhancement should be further addressed as it has been for doping [9], [35], [36], [50] and general illicit drug use [8], [49], [51], [52].

The prevalence estimate for cognitive doping was significantly higher in athletes who used physical enhancers than those who did not. This result is surprising and to the best of our knowledge, there are no comparable data available. This association may represent the previously mentioned general propensity to enhance, or alternatively indicate that physical enhancers act as a gateway for cognitive doping. Further research is needed to disentangle these two possibilities.

In summary, the present study is the first investigation exploring the gateway hypothesis for both physical and cognitive doping. Moreover, randomized response estimates were used for the first time to address a potential association between doping and legal substance use. Furthermore, we highlight that substance use to improve physical performance and substance use to improve cognitive performance are strongly related to each other, opening a new field for future studies. Finally, we want to stress that the results of this study are limited to a specific population, namely the collective of long- and half-distance triathletes. In other populations, for example non-endurance athletes, professional athletes or non-athletes, the association between the two types of enhancement might be quite different.
